# Supplementary material for: The Association between Message Framing and Intention to Vaccinate Predictive of Hepatitis A Vaccine Uptake
Source: Int J Environ Res Public Health. 2024 Feb 10;21(2):207. doi: 10.3390/ijerph21020207 (PMC10888360; doi:10.3390/ijerph21020207)
Supplement: Supplementary file 1 [file ijerph-21-00207-s001.zip › ijerph-2839756-supplementary.pdf]

Supplementary Material

Table S1. Message frame conditions and corresponding information.

Message Frame	Message Outcome	Message Visuals
Gain Frame	Individual	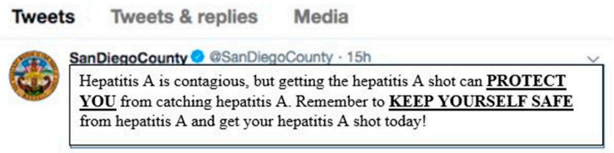
Gain Frame	Collective	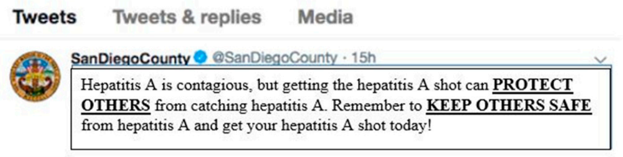
Loss Frame	Individual	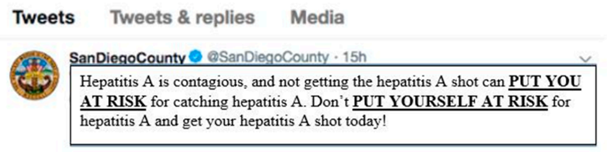
Loss Frame	Collective	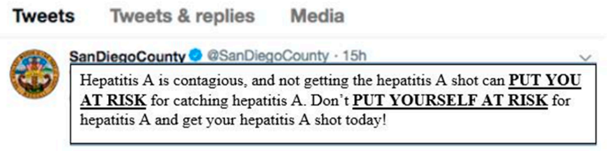
No message (control)	No message (control)	No message (control)

Table S2. List of health behavior constructs assessed throughout the study.

**Message valence**	**Very Negative**	**Somewhat Negative**	**Neutral**	**Somewhat Positive**	**Very Positive**
Thinking back to the tweet you just saw, please indicate how positively or negatively you viewed the message. The message was…	1	2	3	4	5
**Message credibility/likability:** Thinking back to the tweet you just saw, please indicate which adjectives best represent the message.	**Describes tweet very well**				**Describes tweet very poorly**
Accurate	1	2	3	4	5
Believable	1	2	3	4	5
Authentic	1	2	3	4	5
Boring	1	2	3	4	5
Enjoyable	1	2	3	4	5
Lively	1	2	3	4	5
Select 2*	1	2	3	4	5
Pleasing	1	2	3	4	5
Helpful	1	2	3	4	5
Interesting	1	2	3	4	5
**Message perceived effectiveness:** Thinking back to the tweet you just saw, please indicate how much you agree or disagree with the following statements. The message is likely to…	**Strongly Disagree**	**Disagree**	**Neither Agree nor Disagree**	**Agree**	**Strongly Agree**
Persuade someone to get the Hepatitis A shot in the past year	1	2	3	4	5
Convince someone to get the Hepatitis A shot in the past year	1	2	3	4	5
**Theory of Planned Behavior (TPB) – Intention**	**Strongly Disagree**	**Disagree**	**Neither Agree nor Disagree**	**Agree**	**Strongly Agree**
I intend to get the Hepatitis A shot when it is recommended.	1	2	3	4	5
**Health Belief Model (HBM) – Perceived Susceptibility**	**Strongly Disagree**	**Disagree**	**Neither Agree nor Disagree**	**Agree**	**Strongly Agree**
Hepatitis A is a disease to worry about	1	2	3	4	5
Everyone is at risk of getting the Hepatitis A	1	2	3	4	5
Hepatitis A is something I am worried about getting	1	2	3	4	5
I received the Hepatitis A shot in the past year, so I do not need one this year	1	2	3	4	5

I have had the Hepatitis A before so I am no longer at risk	1	2	3	4	5
Select 4*	1	2	3	4	5
**HBM – Perceived Severity**					
People die from Hepatitis A every year	1	2	3	4	5
**HBM – Perceived Benefits**					
The Hepatitis A shot can prevent me from getting Hepatitis A	1	2	3	4	5
I can obtain Hepatitis A from receiving the Hepatitis A shot	1	2	3	4	5
I will be fully protected from Hepatitis A if I get the Hepatitis A shot	1	2	3	4	5
I will be more protected with the Hepatitis A shot versus without it	1	2	3	4	5
**HBM – Perceived Barriers**					
The Hepatitis A shot is dangerous to my health	1	2	3	4	5
I will not get the Hepatitis A shot because I do not know where to get it	1	2	3	4	5
I will not get the Hepatitis A shot because I cannot afford it	1	2	3	4	5
I will not get the Hepatitis A shot because I do not have the time	1	2	3	4	5
Select 5*	1	2	3	4	5
**HBM – Cue to Action**					
I plan to or have already gotten the Hepatitis A shot in the past year	1	2	3	4	5
I would get the Hepatitis A shot if my significant other got the Hepatitis A shot	1	2	3	4	5
I would get the Hepatitis A shot if the new recommended to get the Hepatitis A shot	1	2	3	4	5
I would get the Hepatitis A shot if I saw others online getting the Hepatitis A shot	1	2	3	4	5
I would get the Hepatitis A shot if I saw if I was offered the Hepatitis A shot through my job for free	1	2	3	4	5

*This item was included as an attention check to identify and filter out disengaged respondents.
